# A Prospective Cross-Sectional Study on the Correlation of Adenosine Deaminase and HbA1c With Microvascular Complications in Type 2 Diabetes Mellitus at a Tertiary Care Hospital in Central India

**DOI:** 10.7759/cureus.70732

**Published:** 2024-10-02

**Authors:** Sarang S Raut, Anil Wanjari, Vinit Deolikar, Saket S Toshniwal, Abhinav Kadam

**Affiliations:** 1 General Medicine, Jawaharlal Nehru Medical College, Datta Meghe Institute of Higher Education and Research, Wardha, IND

**Keywords:** adenosine deaminase (ada), biomarkers, diabetic nephropathy, hba1c, microvascular complications, type 2 diabetes mellitus (t2dm)

## Abstract

Background

Type 2 diabetes mellitus (T2DM) is a prevalent chronic condition characterized by hyperglycemia, which can lead to various microvascular complications, including diabetic nephropathy, neuropathy, and retinopathy. Identifying reliable biomarkers for early detection and risk stratification of these complications is crucial for improving patient outcomes. Adenosine deaminase (ADA) and HbA1c have emerged as potential markers associated with immune function, inflammation, and long-term glycemic control. This study investigates the correlation between ADA and HbA1c levels and microvascular complications in patients with T2DM.

Material and methods

This prospective observational cross-sectional study involved 150 patients diagnosed with T2DM, focusing on those with diabetic nephropathy, neuropathy, and retinopathy. Clinical data were collected through patient interviews, clinical examinations, and laboratory tests, including measurements of fasting blood glucose, HbA1c, serum creatinine, ADA levels, and urine protein creatinine ratio (UPCR). Fundus examinations and nerve conduction velocity (NCV) tests were performed to assess diabetic retinopathy and neuropathy, respectively. Data were analyzed using SPSS version 25.0 (IBM Corp., Armonk, New York), with statistical tests to evaluate the correlation between ADA and HbA1c levels and microvascular complications.

Results

The study found a significant correlation between elevated ADA and HbA1c levels and microvascular complications in patients with T2DM. Higher ADA levels were particularly associated with diabetic nephropathy (p=0.003), while HbA1c levels showed a positive correlation with all three complications: nephropathy, neuropathy, and retinopathy. The findings suggest that ADA and HbA1c levels can serve as valuable biomarkers for identifying patients at higher risk of developing these complications.

Conclusion

This study highlights the potential of ADA and HbA1c as biomarkers for early detection and risk assessment of microvascular complications in T2DM patients. Routine monitoring of these markers could improve the management and prognosis of diabetic patients by enabling timely interventions to prevent or mitigate the progression of complications. Further research is needed to explore the underlying mechanisms linking ADA with diabetic complications and to validate its clinical utility.

## Introduction

Type 2 diabetes mellitus (T2DM) is a chronic metabolic disorder characterized by hyperglycemia resulting from insulin resistance and relative insulin deficiency [1]. It is one of the most common non-communicable diseases globally, with a prevalence that continues to rise due to increasing rates of obesity, sedentary lifestyles, and aging populations [2]. The International Diabetes Federation (IDF) estimates that approximately 463 million adults worldwide were living with diabetes in 2019, a number expected to rise to 700 million by 2045 if current trends continue [3]. The chronic hyperglycemia associated with T2DM is a major risk factor for the development of microvascular complications, including diabetic retinopathy, nephropathy, and neuropathy. These complications significantly contribute to morbidity and mortality among diabetic patients and impose substantial economic burdens on healthcare systems [4]. Diabetic retinopathy is a leading cause of blindness among adults, while diabetic nephropathy is the primary cause of end-stage renal disease in developed countries. Diabetic neuropathy, which affects peripheral nerves, is associated with a high risk of foot ulcers and amputations [5].

Monitoring and controlling blood glucose levels through HbA1c is a well-established approach to managing T2DM and preventing its complications. HbA1c reflects average blood glucose levels over the past two to three months and is widely used as a marker for long-term glycemic control. Studies have consistently shown that elevated HbA1c levels are associated with an increased risk of microvascular complications [6]. However, HbA1c alone may not fully capture the complex pathophysiology of T2DM and its complications, necessitating the exploration of additional biomarkers. Adenosine deaminase (ADA) is an enzyme involved in purine metabolism, playing a crucial role in the development of cellular immunity. Elevated ADA levels have been observed in various inflammatory and autoimmune conditions, suggesting a potential link between ADA and the inflammatory processes underlying diabetic complications [7]. Recent studies have indicated that ADA may be involved in the pathogenesis of diabetes and its complications, making it a candidate biomarker for assessing the risk of microvascular complications in T2DM patients [8].

Given the significant impact of microvascular complications on the quality of life and the burden on healthcare systems, identifying reliable biomarkers for early detection and monitoring of these complications is crucial. This study aims to explore the correlation between ADA and HbA1c levels and the presence of microvascular complications in patients with T2DM, with the goal of enhancing the understanding of the pathophysiological mechanisms involved and improving patient outcomes.

## Materials and methods

Study design

This study was designed as a prospective observational cross-sectional analysis to evaluate patients diagnosed with T2DM who presented with microvascular complications. The objective was to systematically observe and analyze various clinical parameters and outcomes related to these complications over a specified period, without modifying the standard care provided to the patients. The study was conducted from December 2022 to December 2023.

Inclusion criteria and exclusion criteria

Strict inclusion and exclusion criteria guided the selection of subjects to ensure the study's validity and reliability. The inclusion criteria were as follows: patients who met the World Health Organization (WHO) criteria for T2DM, were diagnosed with microvascular complications such as diabetic retinopathy, nephropathy, and neuropathy, were above 18 years of age, and provided informed written consent after receiving a comprehensive explanation of the study. Exclusion criteria included patients who did not consent to participate, those diagnosed with type 1 diabetes mellitus or secondary forms of diabetes, patients with chronic alcoholism, those who had undergone anti-tubercular therapy within the last six months, and patients under the age of 18. These criteria were carefully applied to select a study population that would yield accurate and relevant findings regarding the microvascular complications of T2DM.

Method of data collection

Data collection in this study was meticulously planned and executed to ensure accuracy and reliability. The process began with the recruitment of participants who met the inclusion criteria. Upon enrollment, each patient underwent a thorough clinical examination to identify the presence and extent of microvascular complications associated with T2DM. These complications included diabetic retinopathy, nephropathy, and neuropathy, which were diagnosed through specific clinical and laboratory investigations. Clinical data were gathered through detailed patient interviews and examination of medical records. The interviews were conducted to collect demographic information, including age, gender, and duration of diabetes, as well as lifestyle factors such as diet, physical activity, and smoking habits. The medical history was reviewed to document any previous or ongoing treatments, comorbid conditions, and the management of diabetes, including medications and adherence to treatment plans.

Laboratory investigations played a crucial role in the data collection process. Blood samples were collected from each participant after an overnight fast of at least eight hours. These samples were used to measure fasting blood glucose levels and HbA1c, a marker of long-term blood glucose control. Additional blood tests included serum creatinine to assess kidney function, and ADA levels to evaluate immune function and inflammation. Urine samples were also collected to determine the urine protein creatinine ratio (UPCR), a key indicator of diabetic nephropathy.

Specialized tests were conducted to assess the specific complications of diabetes. Fundus examinations were performed using direct ophthalmoscopy to detect diabetic retinopathy, which affects the retina of the eyes. Nerve conduction velocity (NCV) tests were conducted using the UltraPro S100 EMG/NCS/EP (Natus Medical Incorporated, Middleton, US) neurodiagnostic system to diagnose diabetic neuropathy [9]. These tests measured the speed at which electrical impulses traveled through the peripheral nerves, providing crucial information on nerve damage. The data collection process was carefully standardized to minimize variability and ensure the integrity of the data. All clinical examinations and laboratory tests were performed using calibrated instruments and following strict protocols. The blood and urine samples were analyzed within 15 minutes of collection to prevent any degradation or changes that could affect the results. The results of these investigations were meticulously recorded in a structured format, ensuring that all relevant data were captured for subsequent analysis.

Ethical clearance

Prior to the commencement of the study, ethical clearance was obtained from the Institutional Ethical Committee of Datta Meghe Institute of Medical Sciences, Sawangi (Meghe), Wardha DMIMS (DU) IEC/2022/1082, Date 27/06/2022. The study was conducted in accordance with the ethical principles outlined in the Declaration of Helsinki. The ethical committee reviewed and approved the study protocol, ensuring that all procedures adhered to the highest ethical standards.

Informed consent

Informed consent was obtained from all participants before their inclusion in the study. The consent process involved providing participants with detailed information about the study's purpose, procedures, potential risks, and benefits. Participants were given the opportunity to ask questions and were assured that their participation was voluntary, with the option to withdraw at any time without any consequences for their medical care. For participants who were unable to read, the consent form was explained in their native language, ensuring they fully understood the implications of their participation.

Statistical analysis

The collected data were analyzed using statistical software, specifically SPSS version 25.0 (IBM Corp., Armonk, NY, USA). Continuous variables, such as fasting blood glucose, HbA1c levels, and serum creatinine, were expressed as means with standard deviations, while categorical variables, such as the presence of diabetic complications, were presented as frequencies and percentages. The Kolmogorov-Smirnov test was used to assess the normality of the data distribution. Depending on the distribution of the data, appropriate statistical tests were applied: independent t-tests for normally distributed continuous variables, Mann-Whitney U tests for non-normally distributed variables, and Chi-square tests for categorical variables. Correlations between continuous variables were assessed using Pearson or Spearman correlation coefficients. Multiple logistic regression analysis was performed to identify independent predictors of diabetic complications, adjusting for potential confounders. A p-value of less than 0.05 was considered statistically significant for all analyses.

## Results

In this study, the mean age of the participants was 46.80 years, with a standard deviation of 14.91 years. The age range of the participants spanned from 20 to 86 years, as shown in Table 1.

**Table 1:** Distribution of participants with diabetic nephropathy, neuropathy, and retinopathy

Condition	Frequency	Percent
Diabetic nephropathy		
Absent	36	24.0%
Present	114	76.0%
Total	150	100.0%
Diabetic neuropathy		
Absent	28	18.7%
Present	122	81.3%
Total	150	100.0%
Diabetic retinopathy		
Absent	20	13.3%
Present	130	86.7%
Total	150	100.0%

Table 2 indicates that males and females exhibit similar clinical characteristics, with only minor variations. Females tend to have slightly higher mean values for serum ADA levels, HbA1c levels, and serum creatinine, whereas males show higher mean fasting blood glucose levels and eGFR. Although these differences exist, they suggest that both genders generally have comparable profiles in terms of diabetic control and kidney function.

**Table 2:** Demographic and clinical characteristics by gender ADA: adenosine deaminase; eGFR: estimated glomerular filtration rate; UPCR: urine protein creatinine ratio

Variable	Male (mean±SD)	Female (mean±SD)
Age (Years)	46.85±14.52	44.36±8.69
Serum ADA levels (IU/L)	12.54±5.61	14.62±7.24
HbA1C levels (%)	7.54±1.25	7.85±1.34
Fasting blood glucose (mg/dL)	150.75±45.21	142.50±40.18
Oral glucose tolerance test (mg/dL)	250.75±30.12	245.38±28.54
Serum creatinine (mg/dL)	1.52±0.69	1.48±0.87
eGFR (mL/min/1.73m^2^)	87.58±14.85	82.85±13.58
UPCR	3.01±1.98	2.99±1.98

Table 3 reveals that patients aged 50 years and older tend to have higher serum ADA and HbA1c levels compared to those under 50, indicating poorer diabetic control and increased inflammation with advancing age. Additionally, older patients exhibit slightly lower eGFR and serum creatinine levels, suggesting a gradual decline in kidney function as age increases.

**Table 3:** Comparison of clinical characteristics between age groups ADA: adenosine deaminase; eGFR: estimated glomerular filtration rate; UPCR: urine protein creatinine ratio

Variable	Age <50 (mean±SD)	Age ≥50 (mean±SD)
Serum ADA levels (IU/L)	12.75±5.54	14.25±6.35
HbA1C levels (%)	7.45±1.18	7.95±1.32
Fasting blood glucose (mg/dL)	148.25±44.21	144.50±41.18
Oral glucose tolerance test (mg/dL)	248.75±32.12	246.38±29.54
Serum creatinine (mg/dL)	1.85±0.44	1.41±0.67
eGFR (mL/min/1.73m^2^)	88.74±11.85	83.85±10.85
UPCR	2.85±1.98	1.44±0.98

Table 4 shows that patients with diabetic nephropathy, neuropathy, and retinopathy have significantly higher ADA and HbA1c levels compared to those without these complications. This suggests a strong association between elevated ADA and HbA1c levels and microvascular complications, indicating that these biomarkers are valuable in identifying patients at risk for such conditions.

**Table 4:** Comparison of ADA and HbA1c levels in patients with and without diabetic nephropathy, neuropathy, and retinopathy ADA: adenosine deaminase

Diabetic nephropathy	Mean (present)	Mean (absent)	Test statistic	P-value
ADA levels	17.85	15.52	2.235	0.003
HbA1c levels	8.98	9.85		
Neuropathy			3.558	0.001
ADA levels	13.52	14.52		
HbA1c levels	7.75	8.12		
Retinopathy			4.525	0.004
ADA levels	14.52	13.85		
HbA1c levels	10.51	7.84		

Table 5 demonstrates that ADA levels positively correlate with all three microvascular complications, nephropathy, neuropathy, and retinopathy, with the strongest correlation observed in diabetic nephropathy. This indicates that higher ADA levels are significantly associated with these complications.

**Table 5:** Correlation between ADA levels and microvascular complications ADA: adenosine deaminase

Complication	Correlation coefficient	P-value
Diabetic nephropathy	0.424	0.006
Diabetic neuropathy	0.252	0.019
Diabetic retinopathy	0.182	0.039

Table 6 shows that HbA1c levels positively correlate with diabetic nephropathy, neuropathy, and retinopathy, though these correlations are slightly weaker than those with ADA levels. This further underscores the role of HbA1c as a valuable marker for assessing the risk of diabetic complications.

**Table 6:** Correlation between HbA1c Levels and microvascular complications

Complication	Correlation coefficient	P-value
Diabetic nephropathy	0.379	0.013
Diabetic neuropathy	0.243	0.031
Diabetic retinopathy	0.075	0.041

Table 7 reveals a significant association between higher ADA and HbA1c levels and abnormal results in NCV, fundoscopy, UPCR, and eGFR. This suggests that ADA and HbA1c are strong predictors of diabetic complications affecting the nerves, eyes, and kidneys.

**Table 7:** Association of ADA and HbA1c with clinical tests in diabetic complications NCV: nerve conduction velocity; ADA: adenosine deaminase; eGFR: estimated glomerular filtration rate

Test	Chi-square	P-value
NCV vs. diabetic neuropathy	145.58	0.001
Fundoscopy vs. diabetic retinopathy	0.11	0.001
Urine protein creatine ratio vs. diabetic nephropathy	2.541	0.001
eGFR vs. diabetic nephropathy	3.541	0.001

Table 8 shows that patients with abnormal NCV, indicative of neuropathy, have higher HbA1c levels, fasting blood glucose, and UPCR but lower eGFR than those with normal nerve conduction. This suggests that poor glycemic control is associated with impaired nerve function in diabetic patients.

**Table 8:** Demographic and clinical characteristics by NCV ADA: adenosine deaminase; eGFR: estimated glomerular filtration rate; NCV: nerve conduction velocity; UPCR: urine protein creatinine ratio

Variable	Normal (mean±SD)	Abnormal (mean±SD)
Age (Years)	48.84±9.84	43.85±7.84
Serum ADA levels (IU/L)	12.84±4.85	13.52±6.85
HbA1C levels (%)	7.23±1.24	8.05±1.42
Fasting blood glucose (mg/dL)	145.23±42.15	155.36±44.22
Oral glucose tolerance test (mg/dL)	245.67±28.34	252.14±30.15
Serum creatinine (mg/dL)	1.58±0.84	1.12±0.41
eGFR (mL/min/1.73m^2^)	83.58±12.01	75.51±14.51
UPCR	1.05±0.98	2.47±1.25

Table 9 indicates that patients with abnormal fundoscopy findings, indicative of retinopathy, tend to have higher serum ADA levels, HbA1c levels, fasting blood glucose, and serum creatinine. Still, eGFR is lower compared to those with normal fundoscopy results. This suggests that poorer glycemic control and reduced kidney function are associated with retinal complications in diabetes.

**Table 9:** Demographic and clinical characteristics by fundoscopy ADA: adenosine deaminase; eGFR: estimated glomerular filtration rate; UPCR: urine protein creatinine ratio

Variable	Normal (mean±SD)	Abnormal (mean±SD)
Age (Years)	60.52±11.51	58.51±8.21
Serum ADA levels (IU/L)	13.23±6.14	14.82±7.15
HbA1C levels (%)	7.45±1.30	7.95±1.35
Fasting blood glucose (mg/dL)	148.23±44.15	153.36±45.22
Oral glucose tolerance test (mg/dL)	247.67±29.34	250.14±28.15
Serum creatinine (mg/dL)	1.45±0.66	1.84±0.21
eGFR (mL/min/1.73m^2^)	87.85±12.21	80.11±12.52
UPCR	2.99±1.84	2.87±1.52

## Discussion

The findings of this study underscore the significant correlation between elevated ADA and HbA1c levels with microvascular complications in patients with T2DM. The observed correlations provide insights into the potential pathophysiological mechanisms linking these biomarkers with diabetic complications and highlight their relevance in clinical practice. ADA, an enzyme involved in purine metabolism, has been increasingly recognized for its role in immune regulation and inflammation. Elevated ADA levels have been observed in various inflammatory conditions, including diabetes mellitus, where it may contribute to the chronic inflammatory state that characterizes the disease [7]. In this study, higher ADA levels were significantly associated with diabetic nephropathy, neuropathy, and retinopathy, with the strongest correlation observed in nephropathy. This suggests that ADA could be a useful biomarker for identifying patients at risk of microvascular complications, particularly nephropathy. The association between ADA and microvascular complications may be explained by the enzyme's role in modulating immune responses and promoting inflammatory pathways that contribute to endothelial dysfunction, a key factor in developing diabetic complications [10]. Elevated ADA activity has been linked to increased oxidative stress and the production of pro-inflammatory cytokines, which can damage vascular endothelium and impair microvascular function [11]. The findings of this study align with previous research suggesting that ADA is not only a marker of inflammation but also a potential contributor to the pathogenesis of diabetic complications [12].

HbA1c is a well-established marker of long-term glycemic control and is widely used to predict the risk of diabetic complications. The positive correlation between HbA1c levels and microvascular complications observed in this study is consistent with numerous studies demonstrating the relationship between poor glycemic control and the development of diabetic nephropathy, neuropathy, and retinopathy [6,13,14]. The results reinforce the importance of maintaining optimal HbA1c levels to reduce the risk of these complications. Compared to ADA, the slightly weaker correlations between HbA1c and microvascular complications may be due to the multifactorial nature of these complications. While hyperglycemia is a major driver of microvascular damage, other factors such as hypertension, dyslipidemia, and inflammation also play significant roles [15]. This highlights the need for a comprehensive approach to managing T2DM, addressing glycemic control and other modifiable risk factors to prevent or mitigate the progression of complications.

The study also observed gender and age-related differences in ADA and HbA1c levels, with older patients and females showing higher mean values for these biomarkers. These findings are consistent with previous studies that have reported age-related increases in HbA1c levels, likely due to a combination of age-related insulin resistance, longer duration of diabetes, and cumulative exposure to hyperglycemia [16]. The higher ADA levels observed in older patients may reflect an age-associated increase in inflammation and immune activation, further contributing to the risk of microvascular complications [17]. The strong association between ADA and microvascular complications suggests that this enzyme could be valuable to the existing biomarkers used for risk stratification in T2DM patients. Routine monitoring of ADA levels, alongside HbA1c, could help identify patients at higher risk for complications, enabling early intervention and more targeted management strategies. Further research is needed to explore the potential of ADA as a predictive marker for diabetic complications and to investigate the benefits of incorporating ADA measurement into clinical practice.

Limitations

A key limitation of this study is its cross-sectional design, which restricts the ability to establish causal relationships between elevated ADA and HbA1c levels and the development of microvascular complications in T2DM. The study's findings may not be generalizable to all populations, as the sample was drawn from a specific geographic and clinical setting. Further longitudinal studies are needed to confirm these associations and explore their underlying mechanisms.

## Conclusions

The findings of this study underscore the significant correlation between elevated ADA and HbA1c levels and the presence of microvascular complications in patients with T2DM. The strong association observed between these biomarkers and conditions such as diabetic nephropathy, neuropathy, and retinopathy highlights their potential utility in identifying patients at higher risk for these debilitating complications. Elevated ADA levels, in particular, may serve as an important indicator of the inflammatory and immune responses involved in the progression of these microvascular conditions. Consequently, routine monitoring of both ADA and HbA1c levels could play a crucial role in the early detection, prevention, and management of microvascular complications in T2DM, thereby improving patient outcomes. These findings call for further research to explore the mechanistic links and validate the clinical application of these biomarkers in broader patient populations.
